# Integrated Multi-Omics for Novel Aging Biomarkers and Antiaging Targets

**DOI:** 10.3390/biom12010039

**Published:** 2021-12-28

**Authors:** Lei Wu, Xinqiang Xie, Tingting Liang, Jun Ma, Lingshuang Yang, Juan Yang, Longyan Li, Yu Xi, Haixin Li, Jumei Zhang, Xuefeng Chen, Yu Ding, Qingping Wu

**Affiliations:** 1 Guangdong Provincial Key Laboratory of Microbial Safety and Health, State Key Laboratory of Applied Microbiology Southern China, Institute of Microbiology, Guangdong Academy of Sciences, Guangzhou 510070, China; wuleigdim@163.com (L.W.); woshixinqiang@126.com (X.X.); gdim_liangtt@outlook.com (T.L.); yangls8272@163.com (L.Y.); yj18185238563@163.com (J.Y.); 18868006204@163.com (L.L.); xiyu_0604@163.com (Y.X.); 201920146453@mail.scut.edu.cn (H.L.); zhangjm926@126.com (J.Z.); 2 School of Food and Biological Engineering, Shaanxi University of Science and Technology, Xi’an 710021, China; nwu_majun@hotmail.com (J.M.); chenxf@sust.edu.cn (X.C.); 3 Department of Food Science and Technology, Institute of Food Safety and Nutrition, Jinan University, Guangzhou 510632, China

**Keywords:** aging, aging biomarkers, antiaging targets, multi-omics, aging clock

## Abstract

Aging is closely related to the occurrence of human diseases; however, its exact biological mechanism is unclear. Advancements in high-throughput technology provide new opportunities for omics research to understand the pathological process of various complex human diseases. However, single-omics technologies only provide limited insights into the biological mechanisms of diseases. DNA, RNA, protein, metabolites, and microorganisms usually play complementary roles and perform certain biological functions together. In this review, we summarize multi-omics methods based on the most relevant biomarkers in single-omics to better understand molecular functions and disease causes. The integration of multi-omics technologies can systematically reveal the interactions among aging molecules from a multidimensional perspective. Our review provides new insights regarding the discovery of aging biomarkers, mechanism of aging, and identification of novel antiaging targets. Overall, data from genomics, transcriptomics, proteomics, metabolomics, integromics, microbiomics, and systems biology contribute to the identification of new candidate biomarkers for aging and novel targets for antiaging interventions.

## 1. Introduction

In 2019, there were an estimated 702 million people aged ≥65 years according to world population prospects 2019: Highlights, accounting for 9.1% of the world population. The aged population also grows at approximately 3% per year. In addition, human life expectancy rapidly increases, i.e., from 64.2 years in 1990 to 72.6 years in 2019, and is predicted to increase further to 77.1 years in 2050 [1]. Thus, the risk of developing aging-related diseases increases.

Aging is a physiological process in organisms in which multifactorial processes, including genetic factors, external environmental stimuli, and lifestyle factors, determine a progressive decline over time. Environmental factors may have a cumulative and multiple impact on health and longevity. The idea of “healthy lifestyles and environments” comes from the observation of geographical clusters of centenarians around the world, with five identified “longevity hotspots” known as Blue Zones, which are located in Sardinia (Italy), Okinawa (Japan), Loma Linda (California), Nicoya (Costa Rica), and Ikaria (Greece). Thus, their lifestyles and environments are possibly more conducive to longevity than those of the rest of the world. The populations in these areas are characterized by having an active, stress-free lifestyle, strong community bonds, and spirituality. Maybe these are exactly what we in the “non-blue zone” want to learn. It is also subject to regional restrictions such as lifestyle, economic conditions, and geography; they cannot necessarily be broadly extrapolated. However, among tissues and organs, different individuals age at different rates. The aging rate is highly variant, and these specific changes often affect organ functions [2,3]. The aging of the physiological systems and the changes in their functions lead to various chronic diseases and metabolism-related syndromes [4,5].

Therefore, the characterization of aging-related biomarkers is expected to pave the way for the discovery of novel antiaging targets [6]. Understanding the causes of aging and disease and the relationship between the two is important for aging biomarkers that promote the development of geriatrics and clinical translation. To date, there is no accurate independent aging biomarker that can accurately reflect the aging state or aging rate of people. Aging can be characterized by biomarkers [7]. To achieve this goal, studies at the multi-omics level, which integrates epigenomics, transcriptomics, proteomics, metabolomics, and microbiomics data, can provide a more comprehensive overview [8].

Due to the rapid development of bacterial species resource banks and large biological databases around the world, the use of bioinformatics has remarkably improved, and multi-omics methods are the most promising [9]. Currently, various national projects for the biobanking of samples obtained from many people for subsequent omics analysis exist. For instance, biobanks of aging research omics projects include MARK-AGE [10], EUROBATS, and UKBiobank [11]. Similarly, artificial intelligence will greatly deepen our understanding of aging in the near future and transform the most reliable method of assessing biological age into clinical practice. Previously, we have attempted to characterize the gut microbiota of centenarians to determine whether a relationship between the gut microbiota and human longevity exists. Using 16S rRNA gene and metagenomic sequencing methods, we constructed the longest human gut microbiota trajectory of aging and analyzed the composition and function of the gut microbiota in 247 healthy subjects aged 0–110 years. We also conducted in vitro and in vivo experiments in mice.

The multi-omics approach has become the gold standard in different fields of biological sciences. The multi-omics approach can increase the number of identified markers for aging biomarkers with novel insights into aging and of novel targets for antiaging. In this review, we summarize recent studies in the field of aging based on different multi-omics viewpoints, i.e., genomics, transcriptomics, proteomics, metabolomics, and microbiomics. We integrate aging biomarkers of different omics levels to better discover novel targets for antiaging interventions. These promising aging biomarkers could be useful for clinical research.

## 2. The Necessity of Distinguishing Chronological Age and Biological Age

Without a method to assess the personal aging rate, determining preventive interventions for aging is impossible. Aging biomarkers show the changes in the molecules, cells, and organs of the human body with age. Ideally, these biomarkers should slow their progression with age or reverse to a young state [12]. Chronological age represents a person’s actual age and is calculated based on the time elapsed in a person’s life [13]. Biological age refers to an individual’s overall health status at a certain point in time of physiological age. Generally, the environment, diet, life, and psychological factors should be considered. Biological age has been revealed as a better predictor than chronological age, and its measurement can facilitate the assessment of colonoscopy-related colorectal adenoma risk [14,15].

In aging research, it is common to use chronological age. However, due to the heterogeneity of aging, chronological aging is unpractical. There has been a discrepancy between the predictions of biological age and chronological age. Considering the heterochronism of aging, the measurement of biological age becomes complicated, as it involves the calculation of many target molecules that indicate the dynamics of different processes. The panels of biomarkers can act as an integrated tool for measurements. Generally, special indicators are used to accurately indicate biological age. Klemera and Doubal used chronological age as one of the biomarkers, which is the most popular biomarker [16,17]. The principal component analysis method unites equation construction, correlation analysis, and redundancy analysis [18,19]. The main problem with biological age assessment is that the function of chronological age is unknown among the different available measurement methods. Some people believe that it is a very important biomarker [20], while others consider that the aging rate does not need to be measured by chronological age [21].

Advances in artificial intelligence and statistics provide opportunities to accurately estimate biological age. However, they are not fully effective against heterogeneous populations, and there is no clinical certainty. The application of some aging biomarkers from different sources leads to a reduction in the resolution of most biomarkers. Since the ideal biological age estimation method should be comprehensive and complete, we suggest an integrative approach based on multi-omics technologies for aging biomarkers and novel antiaging targets. This multi-omics method is based on the multi-layered organizational logic of life, thus making the prediction of biological age more accurate.

## 3. Multi-Omics for Aging Clocks

### 3.1. Epigenetics Aging Clocks

Biological age estimation based on DNA methylation has been accurately discussed [22,23,24]. As an “age estimator”, the epigenetic aging clock is used to estimate the epigenetic (biological) age of DNA. It also demonstrates that age-related diseases are associated with higher biological age relative to the chronological age. This phenomenon is called epigenetic age acceleration [22].

The discovery of some aging clocks can predict age-related pathologies, such as cancers, heart disease, and diabetes [25]. There are other types of epigenetic clocks from whole blood [26,27,28,29,30], skin [31], and saliva [32]. The aging clock known as DNA methylation GrimAge is an instrument that allows us to view epigenetic acceleration of aging from a new perspective. It can predict the time-to-death and comorbidity count, time-to-cancer, and time-to-coronary heart disease [33]. In a biological aging clock based on DNA methylation, the main indicator of biological age is the methylation of ribosomal DNA exclusively. It can accurately characterize the biological age and show the organism’s response to the treatment of aging and effective antiaging interventions [34].

### 3.2. Transcriptomics Aging Clocks

The positive increase in age reflects faster biological aging. Peters et al. conducted a meta-analysis of 7074 individual peripheral blood samples, in which 11,908 genes were characterized to create age-related predictors. They found that the average absolute error between the chronological age and the predicted age was 7.8 years [35]. Another important study focused on the aging of the transcriptome of skin cells served as a pioneering method for determining the biological age of such datasets [36]. The age was predicted by linear discriminant, and the median absolute error and average absolute error of 4 years and 7.7 years were obtained, respectively [36,37].

The aging rate varies greatly between individuals and groups and will be significantly affected by factors such as genetics, environment, lifestyle, etc. Due to the data type, transcriptome aging clocks have weaker correlations with the chronological age than DNA methylation aging clocks [24,38]. To address this, a standardized cohort is needed. In a study of 6465 individual blood samples collected from 17 datasets, the differences in technical performance had a more significant effect on blood expression profiles than disease and age itself [39]. Then, cross-platform normalization methods, normalization through reference genes, distribution transformation, and quantile normalization were used to successfully eliminate the batch processing effects. A deep neural network was utilized as a predictive index to yield an average absolute error of 6.14 years and a Pearson correlation accuracy of 0.91 [39].

In summary, the biological age prediction technology based on transcriptomics has developed rapidly, and its accuracy level has been continuously improved. Thus, transcriptomics aging clocks will catch up with methylation aging clocks in the near future.

### 3.3. Proteomics Aging Clocks

To help optimize aging clocks and determine the potential novel targets for antiaging interventions, proteomics aging clocks have been systematically reviewed and analyzed. Proteins are studied, because they significantly change their expression levels with age and represent functional products, unlike transcriptome changes, which are not always associated with proteome changes [40,41]. Since previous proteomics studies have used various proteomics techniques, sample sizes and types, and statistical methods, significant differences in the results have been observed. Even when analyzing the same biological sample, the findings can be quite different [42,43].

To achieve these goals, a systematic review of 36 different proteomic analyses was performed, each of which identified proteins that changed significantly with age [44]. There were 32 proteins that have been reported at least five times and 1128 at least twice. Each of these 32 proteins is related to aging and age-related diseases. Furthermore, 1128 common proteins associated with gene regulation, extracellular matrix, and inflammation were analyzed based on bioinformatics enrichment. Finally, a new proteomics aging clock was proposed, which is composed of three or more proteins in the plasma that change with age in different studies. Using a large patient cohort of 3301, the proposed proteomics aging clock was confirmed to accurately predict the age of a person [44].

Another study analyzed 2925 plasma proteins in a cohort of 4263 subjects and developed a new bioinformatics method. This study revealed significant nonlinear changes in the human plasma proteome with age. Changes in the proteome reflect the different biological pathways and reveal the various genome and proteome associations with age-related diseases and phenotypic traits. This new method of studying aging may provide potential novel targets for age-related diseases [45].

### 3.4. Metabolomics Aging Clocks

Hertel et al. [46] proposed the use of metabolomics for biological age prediction, called the “metabolomic aging clock”. They based their analysis on urine data obtained through ^1^H-NMR spectroscopy. The metabolomics aging clock can predict the prognosis of weight loss in bariatric surgery patients and can be applied to other fields of medicine. Similarly, van den Akker et al. [47] developed an innovative, biological age measurement method based on metabolomics and analyzed the ^1^H-NMR serum metabolomics data. To estimate chronological age, they used a linear model trained with metabolomic variables. Finally, they constructed a score reflective of an individual’s biological age called metaboAge and showed that the excess of metaboAge over chronological age corresponded with a poor cardiometabolic health.

### 3.5. Microbiomics Aging Clocks

Using the microbiome aging clock to predict biological age is a relatively new analytical method. However, this method has two problems: one is to find people with similar lifestyles, and the other is to normalize the dataset. Under normal circumstances, the structure and composition of the human gut microbiota will decrease with age; however, the elderly occasionally exhibit a microbiota structure similar to that of adults. The gut microbiome is mainly composed of four phyla: Firmicutes, Proteobacteria, Bacteroidetes, and Actinobacteria [48]. During aging, the relative abundances of *Bifidobacterium*, *Bacteroides*, *Lactobacillus*, *Ruminococcus*, and *Bacillus* decrease, whereas those of *Streptococcus*, *Enterobacter*, *Clostridium*, and *Escherichia* increase [49,50]. The results of studies on aging-related microbial communities are similar to those in microbial communities. In addition to transcriptomics studies, microbiology studies also heavily rely on methodology [51].

In the transcriptional microbiology of aging, the concentration of short-chain fatty acid products in the gut of aging people is low, and it is related to the increase in the number of pathogenic and gas-tolerant bacteria, whose reproduction can lead to malnutrition and age-related diseases [49]. Based on the metagenomic dataset and using deep neural network methods to determine the biological age, including 1673 microbial taxa. An average absolute error of 3.94 years, which is remarkably close to Horvath’s [38] 3.4 years, and an *R*^2^ value of 0.81 were obtained. This is the first study to establish a quantitative model of gut microflora aging [49].

## 4. Multi-Omics Approach for the Discovery of Aging Biomarkers

Aging is the main risk factor for chronic diseases that limits a healthy lifespan. Therefore, the mechanism of aging is a potential therapeutic target. Age correlation analyses involve large amounts of data obtained from various omics analyses, such as genomics (epigenomics), transcriptomics, proteomics, metabolomics, and microbiomics. The main advantages of this method include the analysis of all possible data pertaining to a single person or a large group of people, as well as the common and individual characteristics from a multi-dimensional perspective and the identification of aging markers and novel antiaging targets. Machine learning methods based on deep neural networks are the latest and most complex methods for identifying human aging biomarkers. They can utilize any type of omics data to predict age.

### 4.1. Aging Genomics

#### 4.1.1. Aging Epigenomics

Epigenetics is the study of changes in the biological phenotype without change alterations in the intrinsic genotype [52], and these changes are mainly caused by the environment [53]. The DNAm model is the most studied epigenetic feature [54,55,56]. The epigenetic aging clock is a useful predictor of age-related diseases. Most studies on DNAm patterns analyzed peripheral blood samples and showed that the over- and undermethylation of CpG sites are related to mortality. A total of 353 CpG sites can be used to estimate physiological aging [56]. On the other hand, the immune system status can be characterized by 73 CpG sites [23,24], and 10 CpG sites can be used as predictors of cancer and cardiovascular disease mortality [28]. DNAmGrimAg has been correlated with diseases and can predict mortality [33].

The indicators of epigenetic aging are also related to neurodegenerative diseases. For example, Parkinson’s disease (PD) is associated with the first acceleration of epigenetic aging clocks [57]. Higher epigenetic age (increased DNAmAge) corresponds with a higher risk for cancer and age-related cartilage degenerative diseases [58,59]. Exercise can improve the DNAm of sarcopenia-related genes, in which the epigenetic aging clock is lower [60]. In addition, the epigenetic aging clock increases with BMIs in patients with obesity and metabolic syndrome [61], indicating the relationship between the epigenetic clock and lifestyles.

#### 4.1.2. Aging Gene Expression

Further, overexpression of the Forkhead box O3 gene (*FOXO3*) in model organisms is related to a prolonged lifespan. *FOXO3* overexpression in the adipose tissue of *Drosophila* [62] and mice results in an extended lifespan [63]. Polymorphisms in the *FOXO3* gene in humans are also associated with longevity [64]. Furthermore, the apolipoprotein E gene (*APOE*) encodes a major cholesterol carrier that supports regulation of the cholesterol and lipid metabolism and cell repair [65]. Furthermore, knocking out the tumor suppressor gene *P*53 in mice results in premature aging, organ atrophy, osteoporosis, and a poor antistress response [66].

#### 4.1.3. Telomere-Based Biomarkers

Telomeres are the protective caps of nuclear proteins at the end of eukaryotic chromosomes, composed of repeated TTAGGG. They have the functions of controlling cell and human aging and protecting chromosomes [67]. Telomere length is a typical indicator of biological aging, and telomerase is the main regulator of telomeres [68]. Cell division is accompanied by the shortening of telomere and gradually leads to chromosomal instability with age [69]. In one study involving the systemic knockout of mouse telomerase subunits, a decrease in telomere length, an acceleration of organ dysfunction, and a shortened lifespan were observed [70,71]. The reintroduction of telomerase has great potential for reversing aging [72].

Telomere wear may increase the risk of aging-related diseases [73]. A study on a large population (*n* = 105,539) showed that women have longer telomeres than men and that there are gender-related differences in biological aging [74], which may be due to hormonal differences, such as estrogen levels and the role of the X chromosome [75,76]. There is also a relationship between shorter telomeres and higher mortality [77,78]. Decreased age-related immune surveillance and increased inflammation are associated with the shortening of telomeres and decreased telomerase activity [79]. The telomere length of white blood cells in patients with heart failure was reduced by nearly 40% [80] and, in AD patients, was often shorter [81,82]. Telomere shortening is related to diseases caused by oxidative stress, including Alzheimer’s disease [83], diabetes, and cardiovascular diseases, as well as the proinflammatory cytokine tumor necrosis factor alpha [84,85]. An important factor of aging may be the accumulation of reactive oxygen species (ROS) [86]. Notably, the oxidative stress associated with mitochondria also plays an important role. Particularly, carbonyl cyanide-4 (trifluoromethoxy) phenylhydrazone, the uncoupling agent of oxidative phosphorylation in the mitochondria, can depolarize the mitochondria. Mitochondrial dysfunction can increase ROS production, telomere wear, and genome instability [87]. Moreover, cancer cells can proliferate indefinitely due to their ability to maintain a telomere length [88]. However, the relationship between telomere length and cancer susceptibility remains unclear, because cancer types have distinct characteristics [89]. Additionally, it should be further studied whether telomeres and telomerase can be used as direct biomarkers of aging diseases. Specific drugs or nutraceutical methods that can maintain the great conditions of telomere and telomerase without causing adverse reactions should also be explored [90]. In Table 1, we summarize the main potential aging biomarkers identified in genomics studies.

### 4.2. Aging Transcriptomics

#### 4.2.1. Transcriptomics-Based Biomarkers

Deep changes in the transcription profile occur in human aging processes. Another typical example of agingomics is transcriptomics, which studies mRNA groups, including lncRNAome, circRNAome, and exosomal RNAome. Due to the diversity of the methods and goals in each field, there is no unified way to achieve a clear overall view. Visualizing the complete transcriptome also remains a challenge. Focus on research may affect transcriptomics and assist doctors in selecting appropriate biomarkers from various RNA types [91,92,93,94]. At present, the characteristics of six gene expression markers of cell senescence have been identified by Frenk & Houseley [95].

As a regulator of lipid homeostasis, phospholipid transport, and macrophage activity, ABCG1 mediates the pathway of endothelial cholesterol efflux and protects blood vessels from chronic inflammation. Such alleles usually determine the human lifespan [96,97,98]. A study of the human whole-blood transcriptome including 1016 people aged 70–80 years showed that *BIRC2* is an apoptosis regulator of inflammation, cell proliferation, and mitotic kinase signal transduction and was the most downregulated during aging [99]. In another study analyzing whole-blood samples, aging was positively correlated with the expression of 11 genes, namely *AMZ1*, *MANEAL*, *PARP3*, *KIAA0408*, *ISM1*, *CRIP1*, *NEFL*, *PHLDA3*, *DDB2*, *CHN1*, and *CAPN2*, whereas it was negatively correlated with that of four genes, namely *MXRA8*, *SLC4A10*, *CD248*, and *PLEKHA7* [100].

Five transcriptional biomarkers that can distinguish between <65 years old and ≥75 years old have been accurately determined, thereby demonstrating that transcriptomics can classify the elderly [101,102]. The expression of age-related genes can be used to identify aging biomarkers.

#### 4.2.2. MiRNAs, lncRNAs, and circRNAs-Based Biomarkers

MiRNAs are 21–25 nucleotides in length that are involved in biological regulation processes [103,104]. To identify transcriptome-specific biomarkers, the correlation between microRNA expression profiles and chronological age is analyzed. For instance, the expression of miR-22-3p and miR-28-3p are positively correlated with age, whereas that of miR-425-3p, miR-182-5p, and miR-99b-5p are negatively correlated [92]. MiRNA is related to many diseases, such as cancer [105,106], cardiovascular diseases [107,108], hypertension [109], obesity [110], and diabetes [111]. Multiple studies on monocytes and the serum of long-lived and elderly individuals have revealed age-related miRNAs [112,113,114].

Monitoring the changes in miRNA expression during aging may be useful in detecting promising biomarkers [92]. In sarcopenia, biomarkers such as miR-181a, miR-434-3p, miR-431, miR-29, and miR-126 are involved in IGF-1, senescence, and apoptosis signaling in cells [115]. MiR-19a-3p has been recommended as a biomarker for ischemic stroke, and the gene pathways targeted by miRNAs related to inflammation, coagulation, and platelet activation have been identified [116]. Considering the association between stroke and age and that the elderly population has a higher risk of stroke, the identification of miRNAs can be used for various age-related diseases to subsequently discover biomarkers for disease treatment and prevention [117]. Similarly, human hearing loss is related to the expression of miR-34a and miR-21, which may be potential biomarkers of inflammation [118,119]. MiR455-3p has been proposed as a potential peripheral biomarker for Alzheimer’s disease [120,121]. To elucidate the interaction among miRNA, aging diseases, the aging process, and the underlying mechanisms, there is need for more longitudinal studies and the integration into multi-omics methods.

LncRNAs are ≥200 nucleotides in length that are another type of noncoding RNA [122] and act as signals, baits, and guides during transcription and affect gene expression on different levels, including recombination, transcription regulation, and post-transcriptional modification [123], thereby affecting the length of life and aging. The downregulation of lncRNA induces decreased cell growth and senescence [94]. Telomere-lncRNA can regulate cell telomerase activity during aging [124]. Age-related lncRNA expression disorders may affect neurogenesis and synaptic plasticity by promoting neuropathy via protein aggregation and neurodegeneration [124]. Meg3 has been thought to be related to cardiovascular diseases of aging [125].

CircRNAs are RNA transcripts produced by the reverse splicing of protein-coding exons. These transcripts may serve as useful biomarkers as they accumulate in the brain during aging [126]. CircRNAs can be detected in the blood [127,128], serum [129], and saliva [130]; they are very valuable biomarkers of aging [131,132]. A recent study has shown that, in multiple system atrophy (MSA) patients, circRNAs are upregulated [133]. In Table 2, we summarize the main potential aging biomarkers identified in transcriptomics studies.

### 4.3. Aging Proteomics

#### 4.3.1. Proteomics-Based Biomarkers

Since it is closer to the phenotype than the genome or the transcriptome, the proteome has become an attractive target for researchers studying aging biomarkers. Proteins usually exert direct effects on information processing through signal transduction pathways. Based on a plasma soma scan, 1301 proteins from 120 participants were analyzed, and 10 major proteins related to aging were studied, i.e., GDF15, NPPB, CTSV, EFEMP1, ADAMTS5, CHRDL1, FSHB, MMP12, SOST, and PTN [43]. In a large cohort of 3301 patients, the proteomics aging clock was shown to accurately predict the age of a person [44]. Furthermore, urine tests are another way to use biomarkers to assess the rate of aging. For instance, urine collected from healthy people through the proteome is characterized by its higher abundance of LGGS3BP, MASP2, DNASE1, ANPEP, and IGFBP1 [134].

Proteomics can be used as an effective method to link the genotype and phenotype [135]. The relationship between circulating peptides (such as GDF8 and GDF11 pro-peptides and GDF8 and GDF11 mature proteins) and proteins has been implicated in accelerating aging phenotypes, and they are all involved in the inflammatory process [136]. The results are related to cardiovascular disease [137] and Alzheimer’s disease [138] processes associated with proinflammatory cytokine profiles.

Therefore, proteomics is expected to decipher the aging process. The identified 11 differentially expressed proteins in the elderly may be useful biomarkers and could provide a basis for understanding the molecular mechanisms related to human health and aging [139]. In the plasma proteome of centenarians, the top 10 proteins related to nonhealthy aging (CRTAC1, CDKL1, CD14, and AOPEP) and healthy aging (TGFBI, TAS1R3, IGFAS, CRISP3, and CLEC3B) were revealed. These proteins can be used as aging biomarkers to develop new targets for clinical interventions [140]. The proteomic characteristics of 244 serum samples showed significant differences in the levels of 1312 proteins. Beneficial changes in human gene regulation can also be linked to longevity [141]. Proteomic studies have shown that serine protease inhibitors, SCT1, and GDF15 proteins can be used as biomarkers of aging, and there is an overlap in content considered as effective biomarkers [142]. In particular, GDF15, a mitogen involved in immune response and immune aging, is worthy of further study, because its concentration increases in elderly people regardless of gender or race [143].

Although some achievements have been made in aging proteomics, reliable proteomic biomarkers are still lacking. The main reasons include the accessibility of mass spectrometry technology, sample selection, maintaining the uniformity of preprocessing procedures, and the lack of proteomic heterogeneity and nonspecific circumvention for different populations, genders, and disease processes.

#### 4.3.2. Senescence-Associated Secretory Phenotype-Based Biomarkers

SASP was first proposed by Coppe and colleagues [144]. Senescent cells can produce and secrete some cytokines, including include growth factors, inflammatory factors, and immunomodulators, to positively or negatively affect the surrounding cells and microenvironment [145]. Severe DNA damage can cause continuous DNA damage response signals and trigger SASP [146]. The NF-κB signaling pathway plays an important role in regulating the expression of IL-6 and IL-8 [147]. The activity of NF-κB is enhanced by mTOR activation and p38 MAPK upregulation [148,149], resulting in a potent SASP.

Sirtuins alone or cooperatively participate in mitochondrial function, cell cycle regulation, inflammation, DNA damage repair, and other biological processes, thus affecting the genome stability, inflammation alleviation, metabolic homeostasis, lifespan, and health maintenance [150,151]. Another protein beneficial for longevity and metabolic regulation is AMP-activated protein kinase (AMPK), affecting animal and human lifespans and health [152]. Besides, the overexpression of deacetylase family genes (Sirtuins) extends the lifespans of yeasts, worms, and fruit flies [153]. According to a recent study, Sir2 prolongs the lifespan by maintaining gene silencing during aging [154]. As another example, telomerase repeatedly adds telomere DNA to chromosome ends to counteract telomere shortening associated with the cell cycle [155]. Further, inhibition of the mammalian target of the rapamycin (mTOR) signaling pathway [156] and mTOR regulatory signals has been proposed as a major molecular mechanism that delays aging in various organisms, from yeasts to mammals [157]. Finally, methionine sulfoxide is considered to be a marker of biological aging [158]. Methionine sulfoxide reductase is a specific antioxidant enzyme that removes this modification of proteins and, at the same time, acts as a general cellular antioxidant to scavenge free radicals and protect the cell from biological oxidative stress [159]. In Table 3, we summarize the main potential biomarkers of aging identified in proteomics studies.

### 4.4. Aging Metabolomics

The biomarkers of aging metabolomics are the most useful tools for estimating aging. Metabolomics yields a huge amount of information, especially when it is used in conjunction with other omics approaches. Biomarkers based on metabolomics can simultaneously become the driving forces and signs of aging, which can then reveal the metabolic pathways related to the lifespan [160]. Changes in the metabolic profiles related to age can be quantitatively analyzed, and the analytical techniques for metabolite detection are highly sensitive and specific [161].

Regarding their clinical application, the number of known metabolomics biomarkers of aging is limited [162]. The use of multi-omics aging pipelines with transcriptomics or other types of omics data makes the metabolome itself valuable [163]. It is necessary to mention the “endophenotype” here. The aging process may be accompanied by different genetic, environmental, and disease effects, which may affect the overall transcriptome, proteome, and metabolome profiles. The multi-omics method based on the endophenotype is a more flexible method to detect age-related metabolome changes [164,165].

In a metabolomics and epigenetic analysis investigating aging in the British population, significant multi-directional changes in several metabolic pathways, such as CoA catabolism, vitamin E metabolism, lysine metabolism, tryptophan metabolism, and tyrosine metabolism, were detected [166]. In another study, 2678 plasma metabolites in a cohort of 150 individuals (30–100 years old) were identified [167]. The levels of monoacylglycerides, diacylglycerols, and phosphoserine decreased with age. On the other hand, the product of proteolysis, i.e., l-γ-glutamyl-l-leucine, increased independently of gender during aging. However, the levels of 25-hydroxy-hexanoic acid, eicosapentaenoic acid, and phosphoserine showed a negative trend in the elderly. Therefore, the effect of aging on lipid distribution can be detected [167].

Various molecular mechanisms underpin the genetic factors involved in longevity. The main factors that affect longevity and aging are growth hormone (GH) and insulin/insulin-like growth factor (insulin/IGF-1) pathways [168] in various organisms, from yeasts to mammals (including humans) [169,170]. Nicotinamide adenine dinucleotide (NAD^+^) plays a vital role in mitochondrial electron transport, and it decreases with age [171]. Dietary supplementation with NAD^+^ can help maintain health and extend the lifespan of mice [172,173]. Nicotinamide ribose supplementation can induce muscle and intestinal stem cells in aging mice to rejuvenate [174,175]. However, its potential antiaging effect has to be balanced with the potential tumorigenesis risk [176].Chronic inflammation in tissues is another cause of aging. It is triggered by signaling pathways related to the activity of transcription factor NF-κB [177]. Inhibiting the activity of NF-κB extends the life of fruit flies and mice [178]. Further, the autophagy–lysosomal signaling pathway eliminates toxic and easily aggregating proteins to maintain the normal cell functions in nematodes [179], fruit flies [180], mice [181], and other model organisms, and even human cells, to extend their lifespan.

In addition, individuals with higher levels of advanced glycation end products (AGEs) suffer from oxidative damage, leading to immune aging [182,183]. There are nine differentially expressed metabolites in men and women, which may serve as biomarkers of the aging process [184]. Another study of 44,168 individuals (18–109 years old) from 12 cohorts revealed that the metabolic profiles of polyunsaturated fatty acids/total fatty acids, histidine, and leucine may be indirect predictors of long-term mortality in clinical trials [185]. However, there are many challenges in clinical metabolomics [186]. In Table 4, we summarize the main potential aging biomarkers identified in metabolomics studies.

### 4.5. Aging Microbiomics

The microbiota exists in all parts of the human body, including the gastrointestinal tract, skin, respiratory tract, and so on. However, its abundance varies depending on tissue and organ types. The human microbiota consists of trillions of coexisting microorganisms, including bacteria, protozoa, archaea, viruses, and fungi [187,188]. The temporal change in microbial diversity and composition [189,190] is essential to human development and health [191]. Many factors affect the diversity and stability of the human microbiome, such as diet, genetics, environment, and antibiotics [192]. During aging, changes in physiology, diet, medications, and lifestyle can lead to changes in the gut microbiota [193].

With the rapid development of high-throughput sequencing technologies, several breakthroughs have been achieved by studies on the gut microbiota [194]. Generally, the diversity of the microbiome decreases with age, especially in the elderly [195,196,197]. For example, *Bifidobacterium,* which plays a role in maintaining human health, is an important part of the gut microbiota [197]. However, the abundances of *Bacteroides* and *Enterobacteriaceae* increased [198]. The long-term supplementation of *Bifidobacterium* can enhance the memory of aging rats [199]. The ratio between Firmicutes and Bacteroidetes can be an indicator of metabolic health and decreases with age [200]. According to studies using human and animal models, the composition of the gut microbiota is an important factor related to longevity [201,202]. Some recent studies have shown that the composition of the human gut microbiota is affected by host age, diet, and environment [203,204]. The relationship of the human gut microbiota with metabolic disorders, obesity, inflammatory bowel disease, and infections has also been investigated [205,206]. The gut microbiota can produce various short-chain fatty acids, essential amino acids, peptides, vitamins, and other organic compounds. These microorganisms are also involved in the digestion and absorption of the gastrointestinal tract and regulate immune, metabolism, and other related physiological processes [207,208].

Considering the abovementioned findings, the relationship between the gut microbiota and healthy aging has been investigated [209,210]. In the elderly, the gut microbiota is related to host immune homeostasis caused by inflammation [211], which may lead to diseases and debilitating processes [212,213]. Chronic inflammation, neurodegeneration, and metabolic syndrome are related to inflammatory aging [198,214]. Immune senescence is usually accompanied by upregulation of the inflammatory response. During aging, the continuous imbalance in the gut microbiota leads to an inflammatory response in the intestinal mucosa [215]. Among the elderly, a specific microbiota phenotype has been detected, and the number of bacteria with anti-inflammatory and immunomodulatory effects, including *Bacteroides, Ruminococcus, Faecalibacterium, Parabacteroides*, and *Clostridium,* is reduced, which may promote the development of common diseases and disorders related to aging. [216,217].

In one of our unpublished studies, using high-throughput sequencing tools (16S rRNA gene amplicons and metagenomics), we obtained the longest trajectory of metagenomic changes in the human gut microbiota with age and characterized the microbiome of Chinese centenarians. The gut microbiota of Chinese centenarians was more diverse than that of young people. We observed the enrichment of several potentially beneficial bacterial groups, including those that produce SCFAs, in centenarians. However, some OTUs associated with beneficial bacteria (*Faecalibacterium*) were reduced. Our data suggest that longer lifespans are linked to health-related probiotics in centenarians. The relative abundances of *Akkermansia*, *Lactobacillus,* and *Christensenellaceae* increased in the elderly and centenarian groups. These bacterial families promote immune regulation, defend against inflammation, and promote healthy metabolic homeostasis [218,219]; therefore, they could be markers of the ecosystem of long-lived people.

In addition, we identified the taxonomy of “longevity-related strains” at the genus level and accurately described the functional changes that occur with aging. To validate these metagenomics results, we conducted in vitro screening and in vivo mouse experiments. We observed high oxidoreductase activity in the microbiota of centenarians and concluded that longevity-related strains play an antioxidant role in humans, thus contributing to healthy aging and longevity. In another study on the microbiome of centenarians, decreased concentrations of *Blautia*, *Coprococcus*, *Roseburia*, and *Faecalibacterium* and high concentrations of *Christensenellaceae*, *Akkermansia*, and *Bifidobacterium,* which are associated with immunological and metabolic health, and a significant increase in concentrations of *Desulfovibrionaceae* and *Enterobacteriaceae* were linked to longevity [220].

Combining at least two omics methods (genomics, transcriptomics, proteomics, and metabolomics) in the study of the microbiome advances the discovery of microbiome biomarkers of aging. A study analyzing the metabolites in human blood samples showed that, with age, the bacteria associated with the metabolism of indole and tryptophan significantly decreased in the gut microbiome. However, the downregulation of tryptophan transport and metabolism is essential for cognitive function and T-cell differentiation [221,222,223]. Tryptophan plays a vital role in intestinal immune tolerance and maintaining the balance of the gut microbiota [224]. The enhancement of tryptophan metabolism has been positively correlated with age, which is consistent with the finding that the serum tryptophan level in the elderly is low [225] and, in patients with dementia, is also reduced [226].

In summary, microbiomics is another promising field for diagnosing senile diseases, discovering novel clinical interventions, and establishing biomarkers of aging. However, future studies need to consider the effect of other species, such as archaea, fungi, and viruses, as well as that of the environment and host. These factors play a vital role in the overall regulation. Since aging is a complex and dynamic process, useful biomarkers in one population may not be applicable to different, other populations. Therefore, longitudinal research cohort studies should be conducted. The standardization of sample collection, processing, and data analysis protocols should also be considered. In Table 5, we summarize the main potential biomarkers of aging identified in microbiomics studies.

### 4.6. Early Biomarkers of Aging

Early biomarkers of aging are key, because it is unlikely that even the best antiaging interventions will be effective in aged individuals, and it would be more important to intervene earlier in life. The gut microbiota may influence the physiological mechanisms of a wide range of age-related diseases and biological phenotypes. It is worth noting that, compared with most age-related disease onsets and age-driven health declines, strategies to repair or improve the dynamics of the gut microbial community at or before this life stage may be useful ways to explore the prevention of premature aging, for example, by supplementing probiotics, targeted dietary changes, or vaccines. In addition, some different taxa may require further research on the physiological aging potential of the microbiota in early to mid-adulthood. Future research will verify these findings through richer interference factor controls, larger sample sizes, longitudinal follow-ups, and direct immunological measurements, which will further support the gut microbiota to help in the early detection and prevention of accelerated aging and age-related diseases.

## 5. Integromics and Systems Biology

To promote the multidimensional analysis of data, advanced omics technology is inseparable from advanced omics analytical tools. At present, large-scale, high-quality, and high-throughput data from various omics methods can be efficiently and independently analyzed. However, separate data analysis and interpretation ignore the correlation and biological interference between different omics levels. Therefore, the integration of single-omics methods is essential for an in-depth understanding of the aging process and its mechanism.

Integromics, the comprehensive analysis of different omics data, and systems biology have provided several breakthroughs in the study of aging and antiaging interventions. Together, they have emerged as a more complex statistical method and combine the experimental data obtained in multiple omics methods with computational models to provide a holistic view of the aging landscape [227]. Considering the complexity and heterogeneity of aging, integromics and systems biology not only provide static maps of molecules but are also used to characterize the mutual changes of molecules over time. This helps determine the optimal time point for aging biomarker measurements and specific antiaging drug treatments. Each omics-level biomarker candidate based on integromics and systems biology has biological relevance. Significant biomarker candidates can be preferentially used as biomarkers of aging in medicine and as new antiaging targets.

Currently, data dimensionality reduction and normalization methods, such as a multifactor analysis or partial least square regression analysis, which can identify the main sources of data differences, are used in aging research [228,229]. Similarly, the principal component analysis method decomposes the data into several factors to facilitate the identification of the factor that can best explain the differential phenotypes among aging patients. Other multivariate analysis methods are used to study the overall correlation of multiple variables and, finally, determine the variables that are most likely to shed light on the biological characteristics of specific differences. However, the combined influence of multiple factors and the high variability of a single dataset may cause difficulties in identifying biologically relevant and unrelated molecules [229].

Since aging is a multifactor, complex disease in which multiple physiological processes are regulated simultaneously and change over time, a single regulation is not sufficient to alleviate or reverse this pathological process. Thus, the use of integromics and systems biology to identify biologically interconnected processes in aging, which can be simultaneously regulated by combination therapy, is essential. Integromics and systems biology can also play an important role in personalized therapy. In cancer, multi-omics methods have been used for disease identification and personalized treatment [230,231]. In autism spectrum disorders, along with integration with clinical data, they have been used to accelerate the development of precision medicine and personalized medicine [232]. In addition, building computer models that predict the occurrence of certain diseases based on multi-omics could compare the biomarkers and pathways shared between diseases, thereby paving the way for efficient drug use. In Table 6, we summarize the main potential biomarkers of aging identified in integromics and systems biology studies.

## 6. Conclusions and Prospects

Rapid advances in science and technology have accelerated the arrival of the “omics era”, thereby enabling researchers to collect and integrate data at different molecular levels. The identification of biomarkers of aging and new targets for antiaging interventions is crucial in aging biology and geriatrics. The multi-level information obtained through multi-omics technology contributes to the increased understanding of the mechanisms of aging and provides new opportunities for the diagnosis and treatment of aging and aging-related diseases.

**Figure 1:**
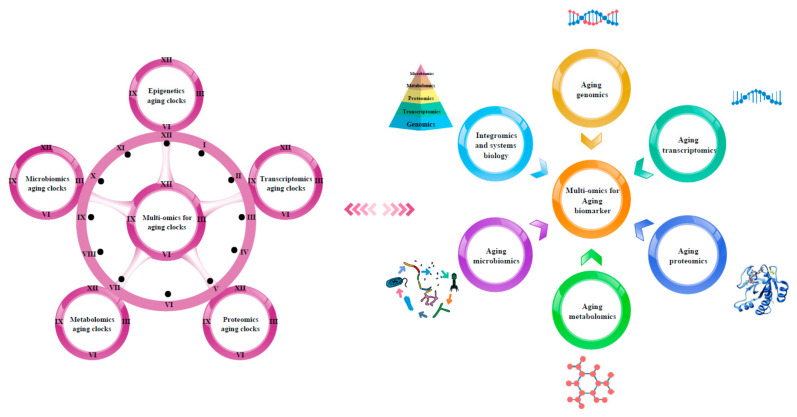
Multi-omics-based technologies for characterizing aging clocks and biomarkers. Aging is a comprehensive process affected by multiple factors that is associated with changes at the molecular, cellular, tissue, and organism levels, thus requiring objective analytical research tools. The integrated multi-omics approach is essential to achieve a comprehensive understanding of the biological mechanisms of aging.

We have summarized the various omics techniques used to characterize aging biomarkers. Each screened biomarker is a promising candidate and can be integrated into an “aging biomarker library” that can serve as a diagnostic and prognostic tool. Here, we mainly categorized them based on the existing biomarkers of aging. We summarized the recent omics methods used to discover biomarkers in genomics, transcriptomics, proteomics, metabolomics, and metagenomics (Figure 1). In the field of geriatrics, discovering new biomarkers from existing datasets and new biological age measurement methods are of great value. At present, a more accurate biological age measurement method based on the aging clock of DNA methylation is needed, which can also be analyzed and evaluated through the transcriptome aging clock. The epigenetic clock from the comparative analysis of actual age and biological age shows that the aging process is inherently related to biological age. In addition, biological age can be measured using transcription profiles. MiRNAs, lncRNAs, and circRNAs contribute to the discovery of novel biomarkers of aging. Proteomics is receiving increasing attention in aging research, because their findings are the most reproducible and the easiest to verify. However, the application of proteomics technology has some limitations, such as high costs and a lack of accurate clinical practice applications. The biomarkers identified in metabolomics and microbiomics studies also have great potential; however, their application in clinical practice is limited by the limited number of longitudinal metabolomics studies available. The comparison of methods from single-omics is the key aspect to better illustrate how integrating these methods will help. This will serve as a synthesis of information rather than just data management. For example, we can compare and analyze the genomics, transcriptomics, proteomics, and metabolomics in microbiology and the substances that appear in the analysis of genomics, transcriptomics, proteomics, and metabolomics in longitudinal populations. For example, we analyzed the correlation between the properties of metabolites regulated by the gut microbiome of healthy and long-lived people and the biomarkers analyzed from blood metabolomics of healthy and long-lived people. This was a good way to show that the integration of what we learned from each method was not just the sum of its parts.

**Figure 2:**
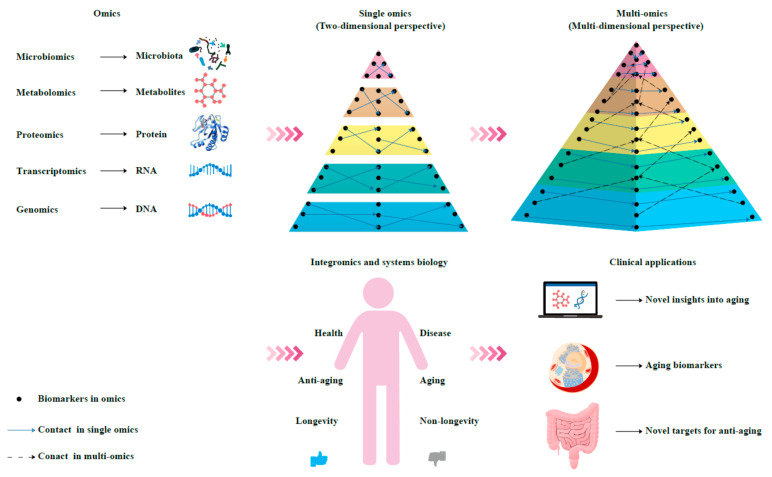
Schematic diagram of an integrated multi-omics approach to the research and application of aging biomarkers. Genomics, transcriptomics, proteomics, metabolomics, and microbiomics enable the high-throughput quantitative profiling of molecules in biological systems to reveal aging-related changes. Combining single-omics data with integromics and systems biology contributes to an increased understanding of the mechanisms of aging and paves the way for the development and utilization of aging biomarkers and novel antiaging targets.

In the context of personalized and precision medicine, multi-omics methods have attracted widespread attention, because they can provide an in-depth understanding of the molecular patterns and cover a wide range of characteristics, such as participating in the metabolic, genetic, and signal transduction pathways of complex aging [233]. Therefore, we suggest that a combination of multiple biomarkers for a comprehensive diagnosis and systematic analysis can objectively characterize the aging process (Figure 2). Integromics and systems biology methodologies can provide insights into organ- and system-specific functions; reflect the phenotype and the processes involved in metabolism, immunity, and structure; and function in different physiological domains and their rates of change in an individual’s lifetime [234]. It can correlate the results at different levels of complexity with clinical profiles. Aging clocks and aging biomarkers and their combinations with multi-omics are usually investigated in experimental studies. However, due to their practicality and feasibility, they are becoming more popular topics in clinical medical research, which advances the knowledge on human aging. Biomarkers have great application prospects in drug target screening, because biomarkers and targets can be the same substance, and they have a high potential for mutual transformation [235]. Methylated aging can evaluate antiaging interventions to develop new types of aging clocks [236]. Biological age is a predictor of mortality in ischemic stroke [237]. In addition, the biological age of the brain can serve as a prognostic tool [238]. Biomarkers are also used for drug discovery and utilization. Designing studies based on biomarkers can help eliminate hidden errors in the treatment process [239]. Recently, a novel biomarker-based miRNA therapeutic strategy for hepatocellular carcinoma (HCC) was successfully applied [240]. Using noninvasive biomarker-based methods, biomarkers contribute to a better understanding of the pathophysiological mechanism of diseases [241]. This review not only focused on single-omics methods to characterize aging biomarkers but, more importantly, integrated multiple omics to evaluate the relevance of these biomarkers and maximized a systematic analysis of the data.

Although multi-omics methods have great potential, limitations and challenges remain. First, omics methods are expensive and require special equipment and highly qualified data analysis personnel. Second, the data quality can be uneven, the data source can be inaccurate, and nonstandard sampling can cause problems in data collection and verification. To date, research platforms and bioinformatics methods for processing large-scale omics data have not yet been standardized. For example, the biggest challenge in metabolomics studies lies in data processing and analysis due to the thousands of metabolites present in biological organisms. Furthermore, the differences among individuals and inconsistent data processing and analysis programs add to this difficulty. Therefore, it is necessary to promote cross-disciplinary efforts and the standardization of procedures to increase the relevance of metabolomics-based strategies in clinical research. Longitudinal cohort studies with large samples should also be conducted. In addition, there is a lack of longitudinal samples and longitudinal studies. In order to delineate the sequence of events, a longitudinal study of the microbiota with age is required. Model organisms with short lifespans and less complex microbiota and established biomarkers of aging make this easier to study. For example, in nematodes, fruit flies, and mice, the integrity of the intestinal epithelium/barrier has been shown to decline with age and is also associated with human aging. Since aging is a complex process that occurs at all levels of biological systems, the impact of antiaging interventions on clinical practice requires a multi-dimensional and systematic approach.

Cellular aging, leading to tissue dysfunction, is widely accepted as contributing to aging and the development of debilitating age-related diseases. Senolytics and bioflavonoids are the key in anti-aging research. Endogenous defenses against ROS include the enzymes superoxide dismutase, glutathione peroxidase, catalase, and peroxiredoxins and the nonenzymatic antioxidants, glutathione, thioredoxin, and uric acid. There are many nonenzymatic endogenous antioxidants. Cofactor coenzyme Q is present in cells and membranes and plays an important role in cellular metabolism and in the respiratory chain. Vitamin A combines with peroxyl radicals, thus preventing lipid peroxidation. Uric acid prevents the lysis of erythrocytes and is also an important scavenger of singlet oxygen. Other small molecular weight nonenzymatic antioxidants include vitamins E and C and many minerals like selenium and zinc. Selenium is the integral part of the antioxidant enzyme glutathione peroxidase. Flavonoids (i.e., flavonols, flavanols, anthocyanins, isoflavonoids, flavonones, flavones, and phenolic acids) act as chelators of transition metal ions involved in Fenton chemistry and ROS scavengers. Bioflavonoids can adjust blood lipids, extend the life of red blood cells, effectively remove free radicals and toxins in the body, and prevent and reduce the occurrence of diseases. Modern pharmacological research shows that curcumin has anti-inflammatory, antioxidant, antitumor, and other pharmacological effects. Resveratrol is a natural antioxidant found in plants. Quercetin may reduce the consumption of glutathione, increase the activity of antioxidant enzymes, and directly or indirectly exert an antioxidant effect in the body after being absorbed by the intestine.

Advances in computer science, including meta-analysis and artificial intelligence, are expected to remarkably increase the speed and efficiency of aging biomarker research [242]. However, before their application in the clinical setting, candidate biomarkers should be verified. This verification process must include larger sample populations. Despite the large gap between the identification of useful biomarkers and their application in clinical practice, the integrated analysis of multi-omics data is a promising tool to identify new candidate biomarkers that could be developed and used to identify pharmaceutical targets and improve human health during aging, thereby advancing our understanding of the pathophysiology of the complex and dynamic process of aging.

## Figures and Tables

**Table 1:** Potential aging biomarkers identified in genomics studies.

Omics	Biomarkers	Function/Application	References
Genomics	DNA methylation aging clocks	Biological age estimation method	[38]
	DNA methylation GrimAge	Been correlation with diseases and can predict mortality	[57]
	DNAm pattern of 353 CpG sites	Estimate physiological aging	[56]
	73 CpG sites	Immune system	[23,24]
	10 CpG sites	Predictor of cancer mortality and cardiovascular disease	[28]
	The increase in DNAmAge	Cancer, age-related cartilage degenerative diseases, and tumor tissues	[58,59]
	Forkhead box O3 gene (*FOXO3*)	Related to prolonged lifespan	[62,63,64]
	The apolipoprotein E gene (*APOE*)	Regulation of the cholesterol and lipid metabolism and cell repair	[65]

**Table 2:** Potential aging biomarkers identified in transcriptomics studies.

Omics	Biomarkers	Function/Application	References
Transcriptomics	Transcriptomics aging clocks	Predictors of age	[35]
	Transcriptome aging of skin fibroblasts	Determining the biological age	[36]
	The number of ABCG1	Determines human lifespan	[96,97]
	BIRC2 gene	An apoptosis regulator of inflammation, cell proliferation and mitotic kinase signal transduction	[99]
	The expression of 11 genes (*AMZ1*, *MANEAL*, *PARP3*, *KIAA0408, ISM1*, *CRIP1*, *NEFL*, *PHLDA3*, *DDB2*, *CHN1*, *CAPN2*)	Positively correlated with aging	[100]
	The expression of 4 genes (*MXRA8, SLC4A10, CD248,* and *PLEKHA7*)	Negatively correlated with aging	[100]
	miR-22-3p and miR-28-3p	Positively correlated with age	[92]
	miR-425-3p, miR-182-5p, miR-99b-5p, etc.	Negatively correlated with age	[92]
	miR-181a, miR-434-3p, miR-431, miR-29, and miR-126	In sarcopenia	[115]
	miR-19a-3p	A biomarker for ischemic stroke	[116]
	the expression of miR-34a	Associated with human hearing loss	[118]
	miR-21	A potential biomarker of inflammation	[119]
	miR455-3p	As early biomarkers of AD	[120,121]
	lncRNAs	Provide different regulatory layers in the cell aging process, which can be used to intervene in this process	[124]
	Downregulation of lncRNA	Lung adenocarcinoma transcript 1 associated with metastasis in proliferating cells induces decreased cell growth	[94]
	Telomere-lncRNA	Can regulate the telomerase activity and survival rate of neural stem cells during aging	[124]
	Age-related lncRNA expression disorders	May affect neurogenesis and synaptic plasticity processes	[124]
	Meg3	Related to cardiovascular aging	[125]
	CircRNAs	May be valuable biomarkers in the aging brain	[126]
	Multiple circRNAs are upregulated	In multiple system atrophy (MSA), which is a sporadic neurodegenerative disease	[133]

**Table 3:** Potential aging biomarkers identified in proteomics studies.

Omics	Biomarkers	Function/Application	References
Proteomics	Proteomics aging clocks	Accurately predict the age of a person	[44]
	GDF15, PTN, ADAMTS5, FSHB, SOST, CHRDL1, NPPB, EFEMP1, MMP12, and CTSV	Related to aging	[43]
	LGALS3BP, MASP2, DNASE1, ANPEP, IGFBP1, etc.	Assess the rate of aging	[134]
	Circulating peptides (GDF8 and GDF11 pro-peptides and GDF8 and GDF11 mature proteins) and proteins	Be related to the accelerated dominant aging phenotype, and they are all involved in the inflammatory process	[136]
	CLEC3B, CRISP3, IGFAS, TAS1R3, and TGFBI	Be related to healthy aging	[140]
	AOPEP, CD14, CDKL1, and CRTAC1	Be related to nonhealthy aging	[140]
	Serine protease inhibitors, SCT1, and GDF15	As biomarkers of aging	[142]
	GDF15	A promising biomarker of aging	[143]
	Sirtuins	Affecting genome stability, inflammation alleviation, metabolic homeostasis, lifespan, and health maintenance	[150,151]
	The NF-κB signaling pathway	Regulating the expression of IL-6 and IL-8	[147]
	AMP-activated protein kinase (AMPK)	Affecting animal and human lifespan and health	[152]
	Telomerase	Counteract telomere shortening associated with the cell cycle	[155]
	Methionine sulfoxide	A marker of biological aging	[158]
	Methionine sulfoxide reductase	Protect the cell from biological oxidative stress	[159]

**Table 4:** Potential aging biomarkers identified in metabolomics studies.

Omics	Biomarkers	Function/Application	References
Metabolomics	CoA catabolism, vitamin E metabolism, lysine metabolism, tryptophan metabolism, tyrosine metabolism, etc.	Related to aging	[166]
	Monoacylglycerides, diacylglycerols and phosphoserine, etc.	Show a decreasing trend with age	[167]
	The product of proteolysis and l-γ-glutamyl-l-leucine	Increases independently of gender during aging	[167]
	25-hydroxy-hexanoic acid, eicosapentaenoic acid, phosphoserine, etc.	Show a negative trend in the elderly	[167]
	Nicotinamide adenine dinucleotide (NAD^+^)	Plays a vital role in mitochondrial electron transport. can help maintain health and extend the life of mice	[171,172,173]
	Higher advanced glycation end products (AGEs) levels	Suffered from oxidative damage, leading to immune aging	[182,183]
	Metabolic profile (polyunsaturated fatty acids/total fatty acids, histidine, leucine, etc.)	May be an indirect predictor of mortality related to clinical trials and medical decision-making	[185]
	Inhibiting the activity of NF-κB	Extends the life of fruit fly and mouse	[177,178]
	The autophagy–lysosomal signaling pathway	Maintain the normal cell functions and extend the lifespan	[179,180,181]

**Table 5:** Potential aging biomarkers identified in microbiomics studies.

Omics	Biomarkers	Function/Application	References
Microbiomics	The abundance of *Bifidobacterium*, *Bacteroides*, *Lactobacillus*, *Ruminococcus*, and *Bacillus* decreased, while the number of *Streptococcus*, *Enterobacter*, *Clostridium*, and *Escherichia* increased	During the aging process	[49]
	The ratio of Firmicutes to Bacteroidetes	Can be used as a criterion for metabolic health, and the ratio will decrease with age	[200]
	*Bacteroides, Ruminococcus, Faecalibacterium, Coprococcus, Parabacteroides, Clostridium, Alistipes*, etc.	Bacteria with anti-inflammatory and immunomodulatory effects	[216,217]
	*Christensenellaceae*, along with *Akkermansia* and *Lactobacillus*	Promote immune regulation, defend against inflammation, and promote healthy metabolic homeostasis	[218,219]
	*Christensenellaceae*, *Akkermansia*, *Bifidobacterium*	Associated with immunological and metabolic health	[220]
	Decrease in *Blautia*, *Coprococcus*, *Roseburia*, and *Faecalibacterium* and significant increase in *Desulfovibrionaceae* and *Enterobacteriaceae*	Linked to longevity	[220]
	*Akkermansia*, *Lactobacillus,* and *Christensenellaceae*	Longevity-related strains play an antioxidant role in humans, which helps achieve healthy aging and longevity	In our study

**Table 6:** Potential aging biomarkers identified in integromics and systems biology studies.

Omics	Biomarkers	Function/Application	References
Integromics and systems biology	The method of comprehensive analysis of different omics data	This method combines experimental data of multiple omics levels with computational models and analyzes them as a whole to identify valuable data	[227]
	Multi-factor analysis or partial least square regression analysis	Can identify the main sources of data differences	[228,229]
	Multi-omics methods	Used for disease identification and personalized treatment in cancer	[230,231]
	Multi-omics and integration with clinical data	Used as a way to accelerate precision medicine and personalized medicine	[232]
